# Plant production of a virus-like particle-based vaccine candidate against porcine reproductive and respiratory syndrome

**DOI:** 10.3389/fpls.2023.1044675

**Published:** 2023-01-24

**Authors:** Jordan T. VanderBurgt, Ondre Harper, Christopher P. Garnham, Susanne E. Kohalmi, Rima Menassa

**Affiliations:** ^1^ Biology Department, University of Western Ontario, London, ON, Canada; ^2^ London Research and Development Centre, Agriculture and Agri-Food Canada, London, ON, Canada; ^3^ Biochemistry Department, University of Western Ontario, London, ON, Canada

**Keywords:** PRRS (porcine reproductive and respiratory syndrome virus), PRRSV, subunit vaccine, virus-like particle, tobacco mosaic virus, plant molecular farming

## Abstract

Porcine reproductive and respiratory syndrome (PRRS) is a disease leading to spontaneous abortions and stillbirths in sows and lowered life quality and expectancy in growing pigs. PRRS is prevalent worldwide and has significant economic impacts to swine industries around the globe. Co-expression of the two most abundant proteins in the viral envelope, the matrix protein (M) and glycosylated protein 5 (GP5), can produce a neutralizing immune response for the virus providing a potentially effective subunit vaccine against the disease, but these proteins are difficult to express. The goal of this research was to display antigenic portions of the M and GP5 proteins on the surface of tobacco mosaic virus-like particles. A modified tobacco mosaic virus coat protein (TMVc) was transiently expressed in *Nicotiana benthamiana* leaves and targeted to three subcellular compartments along the secretory pathway to introduce glycosylation patterns important for M-GP5 epitope immunogenicity. We found that accumulation levels in the apoplast were similar to the ER and the vacuole. Because glycans present on plant apoplastic proteins are closest to those present on PRRSV proteins, a TMVc-M-GP5 fusion construct was targeted to the apoplast and accumulated at over 0.5 mg/g of plant fresh weight. TMVc virus-like particles self-assembled in plant cells and surface-displayed the M-GP5 epitope, as visualized by transmission electron microscopy and immunogold localization. These promising findings lay the foundation for immunogenicity and protective-immunity studies in animals to examine the efficacy of this vaccine candidate as a measure to control PRRS.

## 1 Introduction

One of the major diseases affecting the global swine industry is the porcine reproductive and respiratory syndrome (PRRS) (Holtkamp et al., 2013). This disease causes respiratory symptoms in pigs, as well as stunting of growth and reduced life expectancy. In pregnant sows, PRRS leads to reproductive issues such as spontaneous abortions and stillbirths (Charerntantanakul, 2012). PRRS is caused by the porcine reproductive and respiratory syndrome virus (PRRSV), a small enveloped RNA virus from the family *Arteriviridae* within the order *Nidovirales.* Because PRRS is a viral disease, antibiotics cannot control its transmission. However, a serious consequence of PRRSV infection is secondary bacterial superinfection. Bacterial pathogens such as *Mycoplasma hyopneumoniae* cause more severe disease when PRRSV is present and, for this reason, PRRSV outbreaks are often treated with antibiotics (Glass-Kaastra et al., 2013; Chae, 2016), hence contributing to the spread of resistance to antibiotics genes in both pathogenic and commensal bacteria (Modi et al., 2013; Liu et al., 2019). Vaccines are a preferred option for preventing infection with PRRSV. There are a variety of vaccines currently available against PRRS, however, they are either ineffective at preventing transmission or pose safety concerns (Zuckermann et al., 2007; Okuda et al., 2008; Kimman et al., 2009). There is also clear evidence of recombination between PRRS live attenuated vaccines and field strains (Nielsen et al., 2001; Wang et al., 2019; Eclercy et al., 2019). Therefore, there is currently a need for a safer and more effective vaccine to control this disease.

PRRSV is divided into two distinct species: PRRSV-1 (European) and PRRSV-2 (North American). The two species are very heterogeneous, sharing only around 65% genome sequence identity (Rupasinghe et al., 2022). PRRSV-1 is mainly found in Europe, however, PRRSV-2 is the predominant species circulating around the world (Paploski et al., 2019; Jiang et al., 2020). Because PRRSV-2 is the main cause of global PRRS outbreaks, controlling the spread of this species should be a priority. Therefore, this research focuses on vaccine development against PRRSV-2.

The PRRSV envelope contains several structural proteins including the matrix protein (M) and glycosylated proteins 2-5 (Wissink et al., 2005). The glycosylated protein 5 (GP5) and M are the most abundant in the envelope and form heterodimers through a disulfide bond between their N-terminal ectodomains (Mardassi et al., 1996; Zhang et al., 2021). Several PRRSV structural proteins are known to contain neutralizing antibody binding sites, however, anti-GP5 antibodies appear most efficient at neutralizing the virus and preventing infection (Gonin et al., 1999; Popescu et al., 2017; Stoian and Rowland, 2019). Also, while M contains no neutralizing antibody binding sites, its co-expression alongside GP5 was shown to increase the immunogenicity of GP5 (Jiang et al., 2006). Based on this information, the co-expression of GP5 and M, or at least their ectodomains, is an important pairing for investigating vaccine candidates against PRRS.

A newer method of vaccine production involves the display of antigenic epitopes on the surface of unrelated scaffold proteins, such as virus-like particles (VLPs). VLP-based vaccines have been shown to have stronger interaction with the immune system and elicit better protective immunity than antigenic proteins alone (Bachmann and Jennings, 2010; López-Sagaseta et al., 2016). The tobacco mosaic virus (TMV), a member of the *Tobamovirus* genus, is a virus that targets and infects a wide range of plant species. The TMV virion consists of a long, rigid nanorod structure approximately 300 x 18 nm in size composed of over two thousand identical coat protein subunits (TMVc) surrounding the viral RNA genome (Lomonossoff and Wege, 2018). These nanorods rely on the viral genome for stability, and recombinant production of wild-type TMVc originally resulted in the assembly of only small ring-like structures (Lomonossoff and Wege, 2018). It was later confirmed that repulsive carboxylate groups on TMVc subunits are the cause of this instability, and modifying specific amino acids produced more stable nanorod VLPs (Lu et al., 1996). These modified TMV VLPs can also display foreign peptides on their surface in an organized and repetitive manner (Brown et al., 2013), which can be co-opted for vaccine design.

Plants are an expression system that has emerged in the past thirty years and picked up momentum in the past ten years (MacDonald et al., 2015). Plants are easy and inexpensive to grow, and they act as their own individual bioreactors. While different expression techniques have been developed to allow for recombinant protein expression in all or select plant tissues (Marsian and Lomonossoff, 2016), there has been a shift towards increased use of transient expression in recent years as it can be quickly and easily scaled up to produce large amounts of recombinant proteins (Margolin et al., 2020). Another benefit of plants is that they have more complex protein processing machinery than many other protein production platforms (Strasser et al., 2021). Proteins can be targeted to a wide range of subcellular compartments, allowing for an assortment of possible N-linked glycosylation patterns (Faye et al., 2005; Strasser et al., 2021). Because of this, researchers can mimic the absence, presence, and type of glycan chains on recombinant proteins to their native counterparts.

In this paper, we describe the plant-production and characterization of a modified TMV coat protein displaying a PRRSV epitope. We found that VLPs of various lengths accumulate and surface-display the PRRSV epitope.

## 2 Results

### 2.1 TMVc nanorods accumulate to high levels and assemble into VLP structures

**Figure 1:**
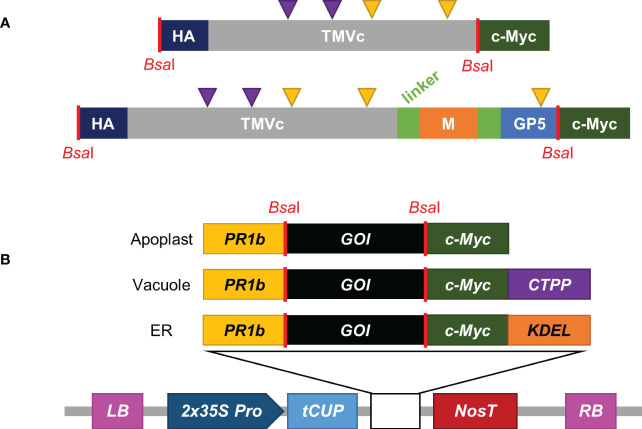
Construct design and pCLGG-X plant protein expression vectors. **(A)** The modified TMVc monomer is shown in grey, with purple arrows representing the altered amino acids (E50Q and D77N). M (orange), GP5 (light blue) and TMVc are separated by (GGS)_4_ flexible linkers (light green). The C-terminal c-Myc tag (dark green) and the N-terminal HA tag (dark blue) are used for detection. Yellow triangles represent potential sites for N-linked glycosylation. Sequence lengths not to scale. **(B)** pCLGG-X plant expression vectors target proteins to the apoplast, vacuole, or ER. All three plasmids contain the double-enhanced *35S* promoter (*2x35S Pro*; dark blue), *tCUP* translation enhancer (light blue) and *Nos* terminator (*NosT*; dark red) between the left and right T-DNA borders (*LB* and *RB*; pink). The signal peptides encoded in the gene constructs include the PR1b signal peptide (yellow), the C-terminal propeptide (CTPP) for vacuolar targeting (purple), and KDEL for ER retrieval (orange). *GOI* indicates the gene of interest. The locations corresponding to *Bsa*I restriction enzyme sites (red lines) used for GoldenGate cloning are noted in both the protein **(A)** and nucleotide **(B)** schematics.

To test if modified TMVc nanorods could be produced in plants, the TMVc sequence from Brown et al. (2013) encoding a two amino acid substitution (E50Q and D77N) was used (**Figure 1A**). This sequence was shown to produce nanorods in *Escherichia coli* but had not been expressed in a plant system.

To determine if the modified coat protein would express well in plants, transient agroinfiltration was performed into *Nicotiana benthamiana* plants. The recombinant protein was targeted to organelles along the secretory pathway (apoplast, vacuole, and ER; **Figure 1B**) because proteins targeted to these subcellular compartments acquire N-linked glycosylation that would later be important when the PRRSV epitope is fused. Because accumulation of some proteins rises steadily with time, while other proteins decrease with time, a time-course was conducted to investigate the accumulation profile of recombinant TMVc for one week after infiltration. This determined the best time to sample the leaf tissue for maximal accumulation of recombinant protein.

**Figure 2:**
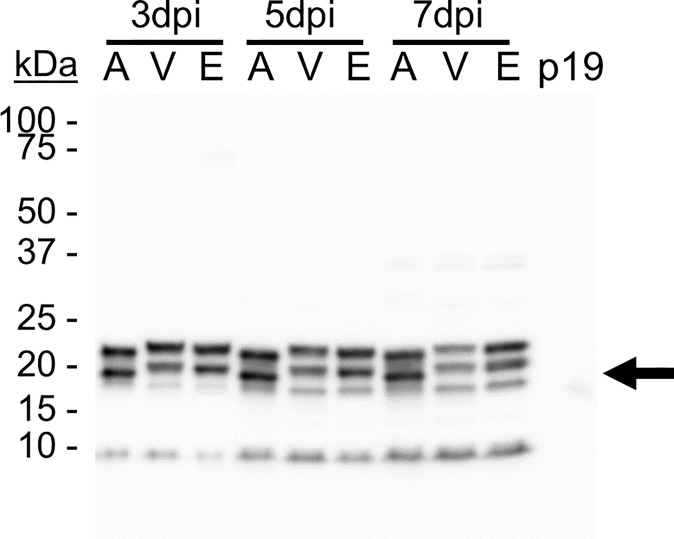
TMVc accumulates in the secretory pathway. Proteins were targeted to the apoplast (A), vacuole (V), and ER (E) and were sampled on 3, 5, and 7 days post-infiltration (dpi). The p19-infiltrated sample acts as a negative control. The black arrow denotes the expected size of unglycosylated TMVc monomer (19.9 kDa). Detection was performed using an anti-HA primary antibody at a 1:5000 dilution. Detection was performed using an anti-HA primary antibody at a 1:5000 dilution, and is representative of two transient expression experiments, and is representative of two transient expression experiments. In each lane 20 µl of total soluble protein were loaded, corresponding to approximately 6.67 mg of leaf fresh weight.

A triplet of bands was detected in most TMVc lanes (**Figure 2**). This can be explained by heterogeneous glycosylation at the two putative glycosylation sites (**Figure 1A**), as determined by NetNGlyc 1.0 server (Technical University of Denmark). The lower band is likely the unglycosylated protein, with the upper bands containing one or two glycan chains. The apoplast-targeted TMVc has only two bands visible, which may be the result of efficient glycosylation as no band for the unglycosylated monomer is detectable. This experiment was repeated, and in both experiments the bands ran slightly lower in the apoplast-targeted samples than in the ER and vacuole samples (**Figure 2**). This is likely due to the KDEL ER retrieval tetrapeptide and the C-terminal vacuole targeting peptide that add amino acids to the mature protein. While no quantification or data analysis was performed for these experiments, visual inspection of band intensity indicated that apoplast targeting was suitable for further analysis. Sampling at 7 days post-infiltration (dpi) was selected to provide more time for the proteins to self-assemble into VLPs.

**Figure 3:**
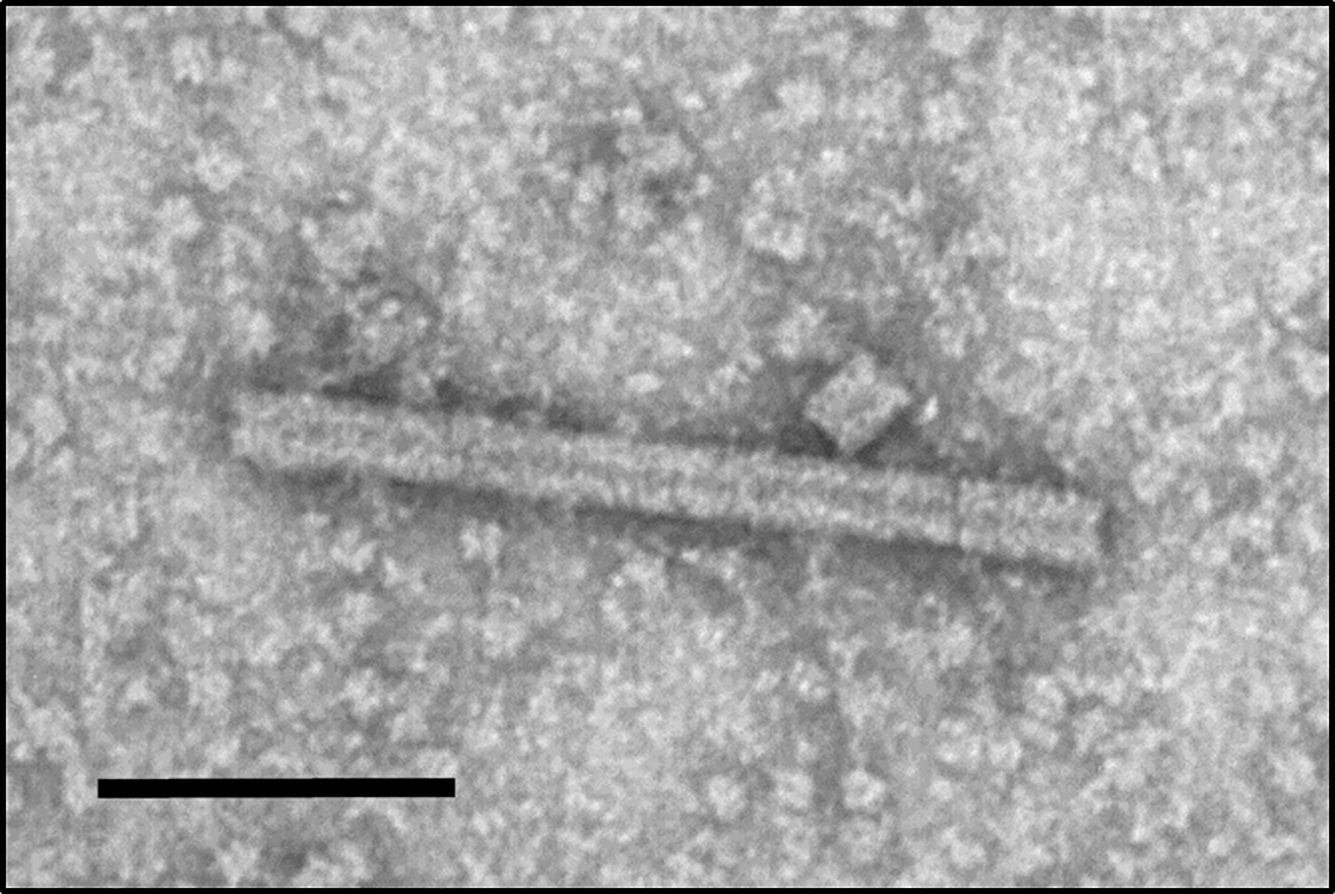
Plant-produced TMVc assemble into nanorod structures. The modified TMVc self-assembles into nanorod structures of varying lengths. Scale bar represents 100 nm. The protein sample was targeted to the apoplast and sampled on 7 dpi. Partially purified VLP extract was negatively stained and examined using a TEM (JEM-1400, JEOL) operated at 80 kV.

To assess the quaternary assembly and nanorod formation of the modified TMVc, VLPs were extracted and partially purified from infiltrated leaf samples then visualized by TEM. Characteristic nanorod structures with 18 nm diameters and varying lengths were observed (**Figure 3**), demonstrating that the modified TMVc protein does assemble into VLPs upon plant expression. The variation in nanorod length was expected as the consistent 300 nm of the native TMV is dictated by the encompassed viral genome. Because the VLPs produced here contain no genetic material, uniform nanorod length could not be expected.

### 2.2 Fusing the PRRSV epitope to TMVc does not affect accumulation or disrupt VLP assembly

The PRRSV GP5 ectodomain was selected for the vaccine epitope because it contains at least one neutralizing antibody binding site within its ectodomain and anti-GP5 antibodies have been found effective at neutralizing this virus, with data suggesting broad-neutralization of heterologous strains (Gonin et al., 1999; Popescu et al., 2017; Stoian and Rowland, 2019). Furthermore, the GP5 ectodomain contains varying numbers of glycosylation sites depending on the viral strain. The PRRSV sequences selected for this study are from the PRRSV-2 reference strain, VR-2332. The GP5 sequence selected for this epitope corresponds to amino acids 30-54 because the first approximately 30 amino acids make up a cleaved signal peptide (Popescu et al., 2017). Also, a neutralizing epitope between residues 37-41 has been previously described (Rupasinghe et al., 2022) so the GP5 ectodomain sequence selected for this research encompasses that epitope, with additional flanking sequences found to be relatively conserved across different PRRSV-2 strains. It was shown that glycan chains at N33 and N51 shield the antibody binding site on GP5 from the immune system (Ansari et al., 2006), while the glycan chain at N44 is important for eliciting antibodies capable of neutralizing wild-type PRRSV (Wei et al., 2012). Therefore, mutations N33A and N51A were introduced in the GP5 ectodomain sequence for improved immunogenicity of this epitope.

Co-expression of M with GP5 was previously shown to amplify the anti-GP5 immune response (Jiang et al., 2006), so pairing the M and GP5 ectodomains together in our designed epitope may have a similar effect. Amino acids 2-18 from M were selected as this corresponds to the entire ectodomain lacking the initial methionine.

The PRRSV M-GP5 epitope was genetically fused to the C-terminus of TMVc as previous work found this terminus is surface-exposed on the nanorods and is amenable to the addition of foreign peptides (Pogue et al., 2007). The M and GP5 ectodomain sequences were separated from each other and the TMVc monomer by flexible (GGS)_4_ linkers (**Figure 1A**).

**Figure 4:**
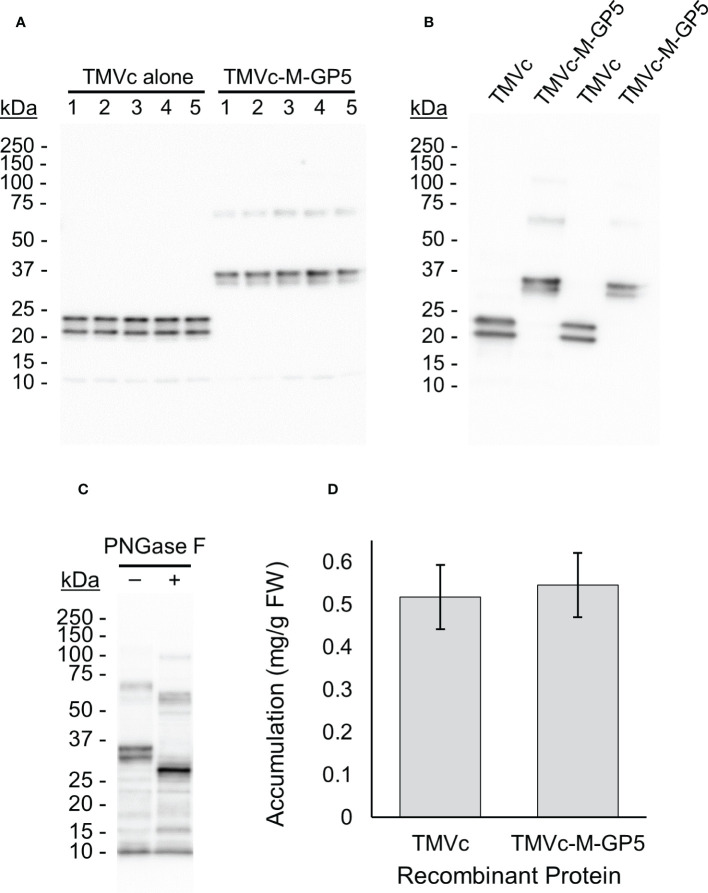
TMVc-M-GP5 accumulates well in plants and is efficiently glycosylated. All proteins were targeted to the apoplast and sampled on 7 dpi. **(A)** The numbers across the top (1-5) identify different plants considered biological replicates. The expected size of the fully glycosylated TMVc and TMVc-M-GP5 monomers are 24.3 kDa and 32.5 kDa, respectively. The 70 kDa band observed for TMVc-M-GP5 may be due to dimerization. A standard curve was generated with known amounts of a protein standard and used for quantification. In each lane 5 µl of total soluble plant proteins were loaded corresponding to approximately 0.5 mg of plant tissue fresh weight. Exact tissue weights were recorded and considered for accumulation values. This blot was probed with an anti-HA primary antibody, and is representative of three transient expression experiments. **(B)** TMVc and TMVc-M-GP5 immunoblot probed with an anti-c-Myc primary antibody. Two replicates are shown for each protein. The same double banding pattern as probing with an anti-HA primary antibody is observed. **(C)** Deglycosylation of TMVc-M-GP5 with PNGase F (+) shows a single band at the expected molecular weight of the unglycosylated protein, compared to the double banding observed for the untreated protein (–). This blot was probed with an anti-HA primary antibody. **(D)** The mean recombinant protein accumulation levels of 15 biological replicates over three independent experiments (5 biological replicates per experiment) are shown in milligrams of recombinant protein per gram of plant fresh weight (FW), with the bars above and below representing 95% confidence intervals. No significant difference in recombinant protein accumulation was observed, as assessed by ANOVA.

Upon transient expression by agroinfiltration, two TMVc-M-GP5 bands were observed on the immunoblot at around 35 kDa and an additional faint band at about 70 kDa (**Figure 4A**). To verify that the double banding does not represent C-terminal cleavage, another sample was immunoblotted with anti-c-Myc antibodies. The same pattern was observed (**Figure 4B**), indicating that both bands represent different glycosylation states of the full length protein. The expected molecular weight of the unglycosylated protein is 25.9 kDa, and that of the fully glycosylated protein is 32.5 kDa. Therefore, it appears as if the dominant protein bands on the immunoblot are from the glycosylated TMVc-M-GP5, and the 70 kDa band may represent a dimer. Deglycosylation analysis on TMVc-M-GP5 resulted in a single band at about 25 kDa confirming that the double-banding pattern is the result of N-linked glycosylation (**Figure 4C**).

To confirm whether the recombinant proteins accumulate in the apoplast, the apoplastic fluid was isolated from leaves infiltrated with TMVc-M-GP5. The recombinant protein was detected in infiltrated leaves compared to a negative control (**Supplementary Figure 1**), indicating localization of the recombinant proteins in the apoplast.

For quantifying soluble protein accumulation, a total of three experiments were run with 5 plants each, resulting in 15 biological replicates. The average accumulation levels of the recombinant protein was not significantly different between TMVc and TMVc-M-GP5 (p-value = 0.59) (**Figure 4D**). Therefore, fusion of the M-GP5 epitope onto TMVc does not appear to alter accumulation upon expression in plants.

**Figure 5:**
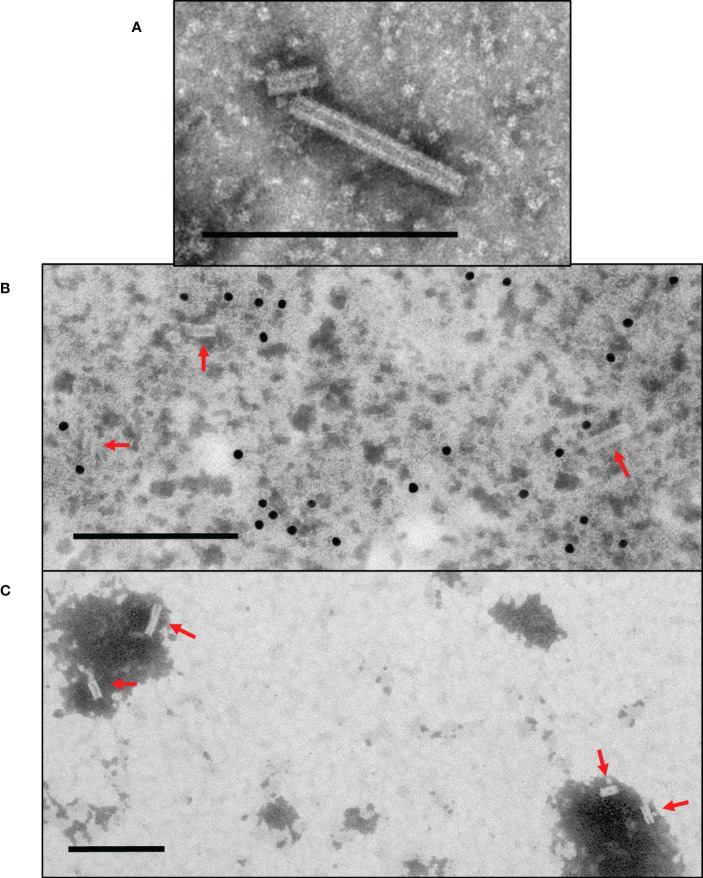
Plant-produced TMVc-M-GP5 assembles into nanorod structures surface-displaying the PRRSV epitope. **(A)** The modified TMVc-M-GP5 fusion protein self-assembles into nanorod structures of varying lengths. **(B)** TMVc-M-GP5 with immunogold localization using anti-c-Myc primary antibodies. Gold particles (10 nm) bound close to the nanorod structures indicate that the PRRSV epitope are displayed on the VLP surface. **(C)** Negative control for immunogold localization of TMVc-M-GP5, using no primary antibody for detection. Nanorods are present but no gold particles are visible surrounding VLPs. Scale bars represent 200 nm, and nanorods are denoted with red arrows. All protein samples were targeted to the apoplast and sampled on 7 dpi. Partially purified VLP extracts were negatively stained and examined using a TEM (JEM-1400, JEOL) operated at 80 kV.

To assess whether fusion of the PRRSV M-GP5 epitope affects VLP assembly, TMVc-M-GP5 samples were prepared and examined using a TEM and nanorods with 18 nm diameters and variable lengths were observed similar to TMVc nanorods (**Figure 5A**). While genetic fusion of the M-GP5 epitope does not inhibit VLP assembly, TMVc-M-GP5 nanorods were generally shorter in length than those of TMVc alone. For example, the average length of 150 TMVc nanorods was 66.42 nm (s.e. 2.79), compared to an average length of 43.48 nm (s.e. 1.32) for 235 TMVc-M-GP5 nanorods. Once assembly of the fusion proteins was confirmed, we assessed whether the PRRSV epitope is displayed on the nanorod surface. Immunogold localization was performed using anti-c-Myc primary antibodies; gold particles were found near the nanorods (**Figure 5B**), suggesting that M-GP5 epitopes are displayed on the nanorod surface. As a negative control, no gold particles were observed when the primary antibody was omitted (**Figure 5C**).

## 3 Discussion

VLP-based vaccines provide safe and effective alternatives to traditional vaccines. Unlike live attenuated vaccines, protein VLPs are unable to replicate or revert to virulence because they lack genetic material (Francis, 2018; Vetter et al., 2018). VLP-based vaccines are also efficient at producing strong and long-lasting immune responses, a benefit they have over many traditional subunit or killed virus vaccine methods (Francis, 2018; Vetter et al., 2018). Because of these reasons, more research has been allocated to studying different VLPs as potential vaccine candidates.

Some recombinantly produced VLPs of pathogenic viruses are now commercially available as vaccines. Two vaccines available for human papillomavirus (HPV) (Gardasil^®^ and Cervarix^®^) are prime examples of this, where the recombinant expression of a major HPV capsid protein results in self-assembling VLPs able to induce protection against HPV infection in humans (Wang and Roden, 2013). More recently, Medicago Inc. (Quebec, QC) produced the first plant-derived vaccine against SARS-CoV-2 to reach clinical trials (Ward et al., 2021). Enveloped VLPs with a modified viral spike protein were produced using transient expression in *N. benthamiana*, resulting in VLPs similar in size and shape to the SARS-CoV-2 virus displaying a high density of spike proteins on their surface. After successful phase II and III clinical trials, this vaccine was approved by Canadian Health Authorities in early 2022 (Dubé et al., 2022).

Unlike HPV and SARS-CoV-2, vaccines for many viruses and bacteria cannot easily be produced by recombinant expression of full or slightly modified viral proteins. In these instances, displaying antigenic peptides from the pathogen on the surface of unrelated scaffold proteins can be an effective alternative. The use of scaffold proteins, such as VLPs, may stabilize viral epitopes and allow for higher accumulation of recombinant protein vaccines (López-Sagaseta et al., 2016). Nanoparticle vaccines can also have adjuvant-like properties due to their size and repetitive epitope-display interacting with the immune system to induce robust immune responses (Bachmann and Jennings, 2010; López-Sagaseta et al., 2016). Therefore, displaying PRRSV epitopes on VLPs could be a promising novel approach to vaccine development for this disease.

In this study, TMVc accumulated to high levels and showed no significant change in these levels when fused to the M-GP5 epitope. These high accumulation levels may be due to correct folding of proteins by chaperones and disulfide bond formation in the ER (Strasser, 2018; Robinson and Bulleid, 2020). Also, the introduction of N-linked glycans in the plant secretory pathway may play a role as glycosylation of certain proteins increases their stability (Solá and Griebenow, 2009). For the TMVc-M-GP5 samples, the faint band running at about 70 kDa may represent a dimer of the recombinant protein. Previous work by Castells-Graells and Lomonossoff (2021) found that plant-produced VLPs also contained dimers and further assembly products, which were completely absent when the same proteins were expressed in insect cells. This is due to some component of the plant extract as insect-produced VLPs incubated in wild-type plant extract developed similar banding patterns. It could not be confirmed, however, whether this occurs within the plant cells, or only in extraction conditions (Castells-Graells and Lomonossoff, 2021).

Wild-type TMVc does not produce nanorod structures when the viral RNA is absent due to repulsive carboxylate groups between the rings of the helix, and while these carboxylate groups play an important role in the assembly and disassembly of the native virion (Lu et al., 1996; Brown et al., 2013) they serve no purpose in displaying vaccine epitopes on TMVc VLPs. Therefore, a modified TMVc sequence was used in this project containing a two amino acid substitution (E50Q and D77N) to allow self-assembly into stable nanorod structures even in the absence of RNA, and these modified TMVc nanorods were able to display foreign peptides on their outer surface (Brown et al., 2013). The current work is the first time this modified TMVc sequence was expressed recombinantly in plants. Our results show that nanorod VLPs can form, in accordance with the previous work from *E. coli*. We have also demonstrated that the PRRSV M-GP5 epitope can be displayed on the outer nanorod surface. While nanorod lengths were shorter on average for the fusion protein, VLP morphology and assembly was not impacted to the extent reported by others (Frolova et al., 2010).

Viral diseases are one of the major hurdles facing the livestock industry, especially because viruses cannot be controlled by antibiotics. Interestingly, PRRSV infection may result in antibiotic treatment due to opportunistic bacterial infections that often accompany this virus (Glass-Kaastra et al., 2013; Chae, 2016). High usage of antibiotics for a wide range of purposes including veterinary medicine has led to the growing issue of antimicrobial resistance, where some disease-causing bacteria are unaffected by most or all of the antibiotics used in their treatment (Liu et al., 2019; Pinto Ferreira et al., 2022). Employing vaccines to control diseases like PRRS would allow for decreased transmission rates, and therefore minimize the use of antibiotics on farms (Pinto Ferreira et al., 2022).

While there are many ways to design and produce vaccines, keeping production costs low is essential for veterinary medicine (Schillberg and Finnern, 2021) as livestock farmers are unlikely to purchase expensive medications for their animals (MacDonald et al., 2015). High accumulation levels of recombinant proteins are a major factor in limiting the production costs. For industrial production of plant-produced pharmaceutical proteins for human use, an unofficial threshold of 1% of total soluble protein, or approximately 0.1 mg/g of recombinant protein per fresh plant weight, is used to evaluate economic viability (Rybicki, 2009). Next to accumulation levels, working with insoluble proteins requires more laborious and costly unfolding and refolding steps. Therefore using soluble proteins, which tend to be correctly folded, is preferred. In the current study, we found high soluble accumulation of TMVc-M-GP5 upon plant-production, with accumulation being over 5x the unofficial industry threshold.

Plant-produced vaccines have the potential to be administered orally to livestock, which provides several benefits over injection. One benefit is avoiding the need to purify the recombinant proteins because the plant tissue can be lyophilized and fed to the animals (Chan and Daniell, 2015; Topp et al., 2016), thereby greatly reducing costs (Schillberg and Finnern, 2021). A second benefit is the technical ease of oral administration, where lyophilized plant tissue could be mixed into livestock feed as opposed to injection of the vaccine by a veterinarian (Chan and Daniell, 2015). This would make it difficult to know the exact dose each pig receives, however, regulations for veterinary medicine are not as strict as for human vaccination and a broader dosage range is acceptable (Topp et al., 2016). A third benefit of oral administration is the direct delivery of the vaccine to the intestinal mucosa, improving the resulting IgA titers (Topp et al., 2016). Unlike parenteral administration which tends to result in higher IgG production and lower IgA levels, oral administration of vaccines can directly stimulate high IgA production in mucosal tissues of the intestinal tract (Topp et al., 2016). Stimulating IgA production in certain mucosal tissues, such as the digestive tract, results in raised specific IgA levels at other mucosal tissues, like the respiratory tract (Hosseini et al., 2015). Because the PRRSV infects at mucosal tissues (Pileri and Mateu, 2016), IgA antibodies are likely better suited for interrupting initial infection of the pigs (Kolotilin et al., 2014; Topp et al., 2016).

Another major benefit of using a plant expression system is the variety of post-translational modifications they can add to proteins. One modification of particular interest, especially for this project, is N-linked glycosylation. Plant and mammalian cells both allow for N-linked glycosylation of proteins. The PRRSV is secreted in pig cells, and acquires the complex N-linked glycan chains characteristic of the mammalian late Golgi (Li et al., 2015). In plants, the complex N-linked glycan chains on apoplast-targeted proteins are the closest to those from the mammalian late Golgi (Margolin et al., 2020; Strasser et al., 2021).

While similar, plant-specific complex glycans decorating apoplast-targeted proteins receive plant-specific β1,2 xylose and α1,3 fucose, compared to the α1,6 fucose and terminal sialic acid sugars found on secreted mammalian proteins (Gomord et al., 2010; Margolin et al., 2020). Because of this, plant-specific sugars may be differentially recognized by the immune system. Early concerns that such plant-produced proteins could cause allergic reactions if injected have been disproved (Margolin et al., 2020) and this would not be a concern for oral administration as animals ingest glycosylated plant proteins regularly with no ill effects. The proteins may, however, be more easily identified as foreign and therefore receive more attention from the immune system (Gomord et al., 2010). For many therapeutic proteins this would be undesirable as their intended purpose does not involve the immune system and being recognized as foreign could increase their turnover rate in the body (Gomord et al., 2010; Margolin et al., 2020). For vaccines, this improved recognition by the host immune system may actually serve to benefit the immunogenicity of the proteins (Gomord et al., 2010). In this study, the plant-produced recombinant proteins are being efficiently decorated with N-linked glycans important for the M-GP5 epitope’s immunogenicity.

Moving forward, animal trials must be conducted to examine the immunogenicity of this particulate vaccine candidate. Adjuvants are routinely used in conjunction with vaccines; however, VLP-based vaccines already show improved interaction with the immune system due to their particulate nature and multivalent display of antigenic epitopes, therefore adjuvants may not be required (Zhao et al., 2014; López-Sagaseta et al., 2016). While there is much work yet to be completed before the vaccine candidate produced here can be used in the field to control PRRS, this work provides a promising foundation for further study. It also adds to the ever-growing evidence that producing protein-based pharmaceuticals in plants is an innovative alternative to more traditional approaches.

## 4 Materials and methods

### 4.1 Construct design and cloning

The *TMVc* sequence was based on the entire TMVc monomer from Brown et al. (2013), containing two modifications compared to wild type (E50Q and D77N). For the fusion protein construct, the PRRSV epitope was composed of amino acids 2-18 of the M protein (accession number: AAO13197.1) and amino acids 30-54 of GP5 (accession number: AAO13196.1) from the PRRSV strain VR-2332. The *GP5* sequence also encoded a two amino acid substitution (N33A and N51A) described by Ansari et al. (2006). The *M* and *GP5* sequences were separated from one another, as well as from *TMVc*, by flexible (GGS)_4_ linkers. Both gene constructs were synthesized by Bio Basic Inc. (Markham, Canada) and included sequences for an N-terminal HA tag and flanking *Bsa*I sites for GoldenGate cloning.

Both genes were cloned into in-house pCLGG-X plant expression vectors targeting proteins to the apoplast, vacuole, or ER using GoldenGate Cloning methodology (Marillonnet and Werner, 2015). All three of these vectors contain a double-enhanced *35S* promoter (Kay et al., 1987) and tobacco cryptic upstream promoter translational enhancer (*tCUP*) (Wu et al., 2001) for high expression, the *Nos* terminator (Bevan et al., 1983), and a *c-Myc* tag for detection and purification of the encoded proteins. A PR1b signal peptide from tobacco is also encoded in all three vectors for targeting proteins to the secretory pathway (Cutt et al., 1988). The ER vector encodes a KDEL tetrapeptide for retrieval of the proteins to the ER (Pelham, 1990), and the vacuole targeting vector contains the coding sequence for a C-terminal propeptide (CTPP) vacuolar sorting signal from the tobacco chitinase gene (Vitale and Raikhel, 1999). The resulting plasmids were transformed into *Agrobacterium tumefaciens* EHA105 cells (Hood et al., 1993). Plasmids were isolated from *Agrobacterium* cultures and verified by sequencing.

### 4.2 Plant growth and care

Wild-type *N. benthamiana* plants were grown at 22°C with 65% humidity on a light:dark cycle of 16:8 h in a walk-in growth chamber. Plants were grown in 4” pots with PRO-MIX BX soil, and were watered as necessary using 20:8:20 fertilizer (N:P:K) at 0.25 g/L of water.

### 4.3 Recombinant protein expression

Infiltrations into *N. benthamiana* leaves were performed as previously described (Miletic et al., 2016). Briefly, *Agrobacterium* cultures containing the genes of interest as well as *p19*, a suppressor of posttranscriptional gene silencing from the *Cymbidium* ringspot virus (Silhavy et al., 2002), were co-infiltrated into the abaxial surface of 6-8 week old *N. benthamiana* leaves. *Agrobacterium* containing *p19* alone was used as a negative control.

For the time course with TMVc, 3 leaves on 5 plants were infiltrated with the apoplast, vacuole, and ER targeted constructs. One leaf disc was sampled from each infiltrated area on 3, 5, and 7 dpi with samples pooled across all 5 plants each day. This transient expression experiment was repeated twice. For the fusion construct analysis, 3 leaves on 5 plants were infiltrated with the apoplast-targeted TMVc and TMVc-M-GP5 fusion constructs. For sampling, one leaf disc was taken at 7 dpi from each infiltrated area with samples pooled by plant. This experiment was repeated three times to allow for 15 biological replicates per protein. All plant tissue samples were weighed, flash-frozen in liquid nitrogen, and stored at -80°C until processed.

### 4.4 SDS-PAGE and western blotting

Total soluble proteins were isolated from the plant tissue as described by Miletic et al. (2016). The proteins were transferred to a polyvinylidene difluoride (PVDF) membrane and detected with mouse anti-hemagglutinin (HA) primary antibody (Sigma-Aldrich Cat. No. H3663) or mouse anti-c-Myc primary antibody (Genscript Cat. No. A00864), anti-mouse horseradish peroxidase-conjugated secondary antibodies (Bio-Rad #1706516), and Clarity Western ECL Blotting Substrates (Bio-Rad Cat. No. 1705061). Proteins were visualized on the MicroChemi 4.2 (DNR Bio-Imaging Systems Ltd.) and quantified compared to a standard curve of known concentrations of a protein standard using GelQuant software (DNR Bio-Imaging Systems Ltd.). The protein standard is a synthetic protein designed with multiple detection tags.

### 4.5 Deglycosylation analysis

TMVc-M-GP5 plant extract was deglycosylated using PNGase F (New England BioLabs Cat. No. P0704L) as per the manufacturer’s instructions, with samples incubated at 37°C for 60-90 minutes. The negative control consisted of the same TMVc-M-GP5 plant extract lacking the PNGase enzyme. The samples then underwent SDS-PAGE and Western blotting for visualization.

### 4.6 Apoplastic fluid isolation

Apoplastic fluid isolation was performed as previously described (Hu et al., 2021). Briefly, infiltrated *N. benthamiana* leaves were sampled between 5-7 dpi. The leaves were submerged and vacuum infiltrated in an apoplastic fluid isolation buffer (20 mM MES, 2 mM CaCl_2_, 0.1 M NaCl, pH 6). The external surfaces of the leaves were dried, and the leaves were placed into a 30 ml syringe inside a 50 ml tube. The samples were centrifuged at 700 x *g* for 45-60 minutes at 4°C. The resulting solutions were collected.

### 4.7 Statistical analysis

To compare the soluble protein accumulation levels of TMVc and TMVc-M-GP5, the values were calculated in mg/g of fresh plant tissue by considering the quantification values from the blots, the volume of plant protein extraction buffer used, the mass of plant tissue per sample, and dilution of samples loaded on gels (i.e. volume of extract loaded per lane). The resulting soluble protein accumulation data (n = 15) was examined using R 4.0.3 (R Core Team, 2020). The analysis began with checking the data for outliers, zero inflation, and balance of the categorical variable. A linear model was used and validated by checking the residual distribution and residuals versus fitted values. Finally, an ANOVA was performed to assess significance in the data.

### 4.8 Transmission electron microscopy and immunogold localization

For visualization of the nanorods using transmission electron microscopy, partial purification of the plant extract was performed as previously described (Dai et al., 2020). Briefly, infiltrated plant tissue was ground in a mortar and pestle with 150 µl of potassium phosphate buffer (pH 7.0) containing 0.1% β-mercaptoethanol per 0.1 g plant leaf tissue. The homogenate was centrifuged at 12,000 x *g* for 5 minutes at 4°C, and the supernatant was filtered through a 100 µm nylon cell strainer. After adding one-third volume of chloroform, the samples were vortexed at maximum speed for 1 minute. The samples were centrifuged at 12,000 x *g* for 10 minutes at 4°C, then the aqueous phase was taken and centrifuged again for 30 minutes. The aqueous phase containing the final VLP preparation was then collected.

To examine nanorod assembly, the partially purified VLP preparation was spotted onto formvar carbon-coated copper grids and negatively stained with 2% phosphotungstic acid (PTA). The grids were examined on a TEM (JEM-1400, JEOL Ltd.) operated at 80 kV. For immunogold localization, grids were spotted with the VLP preparation then blocked with 0.3% bovine serum albumin (BSA) for 15 minutes. The grids were incubated with a 1/650 dilution of mouse anti-c-Myc primary antibody (GenScript A00864) for 1 hour. After 5 washes with 0.03% BSA, the grids were incubated for 1 hour with a 1/15 dilution of 10 nm colloidal gold-conjugated goat anti-mouse secondary antibody (Electron Microscopy Sciences Cat. No. 25129). The grids were washed 5 times with 0.03% BSA, then 3 times with distilled water. Finally, the grids were negatively stained with 2% PTA and examined on the JEM-1400 (JEOL Ltd.) TEM.

## Data availability statement

The original contributions presented in the study are included in the article/**Supplementary material**. Further inquiries can be directed to the corresponding author.

## Author contributions

JV designed the constructs, carried out the experiments, conducted the analysis and wrote the manuscript. OH identified the M and GP5 ectodomain sequences, CPG conceptualized the epitope, SEK co-supervised experimental work, and RM conceptualized the study and edited the manuscript. All authors contributed to the article and approved the submitted version.
