# Supplementary material for: Plant production of a virus-like particle-based vaccine candidate against porcine reproductive and respiratory syndrome
Source: Front Plant Sci. 2023 Jan 24;14:1044675. doi: 10.3389/fpls.2023.1044675 (PMC9902946; doi:10.3389/fpls.2023.1044675)
Supplement: Supplementary file 1 [file DataSheet_1.pdf]

Supplementary Table 1. Amino acid sequences for recombinant proteins. Modified TMVc monomer sequences shown in grey. The HA tag is dark blue, and the c-Myc tag is green. The M ectodomain sequence is yellow, and the GP5 ectodomain sequence is light blue. Flexible linkers are shown in pink.

Construct	Sequence
TMVc	YPYDVPDYASYSITTPSQFVFLSSAWADPIELINLCTNALGNQFQTQQARTVVQR QFSQVWKPSPQVTVRFPDSDFKVYRYNAVLNPLVTALLGAFDTRNRIIEVENQAN PTTAETLDATRRVDDATVAIRSAINNLIVELIRGTGSYNRSSFESSSGLVWTSGPAT EQKLISEEDL
TMVc-M-GP5	YPYDVPDYASYSITTPSQFVFLSSAWADPIELINLCTNALGNQFQTQQARTVVQR QFSQVWKPSPQVTVRFPDSDFKVYRYNAVLNPLVTALLGAFDTRNRIIEVENQAN PTTAETLDATRRVDDATVAIRSAINNLIVELIRGTGSYNRSSFESSSGLVWTSGPAT GGSGGSGGSGGSGSSLDDFCHDSTAPQKVGGSGGSGGSGGSNASADSSSHLQLI YNLTLCELAGTDEQKLISEEDL

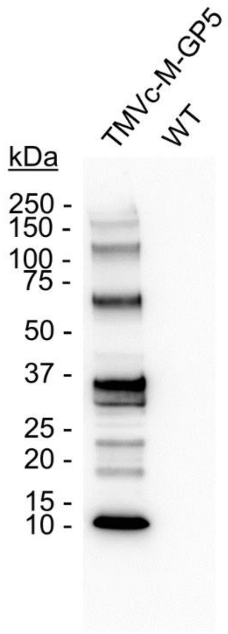

Supplementary Figure 1. TMVc-M-GP5 accumulates in the apoplast. TMVc-M-GP5 is present in the apoplastic fluid of infiltrated plant leaves, compared to equivalent apoplastic fluid from a wild type (WT) leaf tissue control. This blot was probed with an anti-HA primary antibody. In each lane 8 µl of isolated apoplastic fluid was loaded.
